# Impact of tobacco habits on poor oral health status among bone-factory workers in a low literacy city in India: A cross-sectional study

**DOI:** 10.1371/journal.pone.0299594

**Published:** 2024-04-17

**Authors:** Naved Alam, Warisha Mariam

**Affiliations:** 1 Department of Public Health, BRAC JPGSPH, BRAC University, Dhaka, Bangladesh; 2 Division of Immunization, Ministry of Health & Family Welfare, New Delhi, India; Universidade Estadual do Sudoeste da Bahia - Campus de Jequié: Universidade Estadual do Sudoeste da Bahia - Campus de Jequie, BRAZIL

## Abstract

Oral health is a vital indicator of well-being that is influenced by various habits and lifestyles of individuals. Oral diseases are the bottleneck in the effective control of non-communicable diseases (NCDs) due to chronic in nature and reciprocal relationship as sharing the common risk factors and habits such as sugar, tobacco, and alcohol consumption that increase the risk of developing various inevitable diseases. However, there is a lack of literature highlighting the relationship between risk factors for oral diseases and general health among individuals. This cross-sectional study was carried out among 500 study participants aged 20 to 64 years who gave written informed consent and were recruited by Multistage Stratified Cluster Sampling technique among workers in five bone factories, working for at least one year since January 2001 to March 2022 in Sambhal city, Uttar Pradesh. WHO-Basic Oral Health Survey-1997 was used to record the data regarding sociodemographic and oral health status variables. We used the modified WHO-STEPWISE pre-structured questionnaire to record tobacco consumption habits and oral health-seeking behavior. We scheduled a clinical intra-oral examination to record the Decayed Missing Filled Teeth (DMFT) index and the interview on the premises of five bone factories. Among the 500 bone-factory workers, the total number of males was 342 (68.40%) and 158 (31.60%) were females. The mean age (Standard Deviation) was 33.18 (10), and the mean DMFT score of factory workers was 2.84 (3.12). Production workers had the highest mean DMFT score of 4.60 (3.25). More than half of the factory workers (53.2%) were tobacco users. Tobacco users were 3.52 times more likely to have a severe DMFT index. Most common pre-cancerous lesions were oral submucous fibrosis and leukoplakia. Compared to non-tobacco users, mild tobacco users have 6.80 folds higher odds of oral lesions. Tobacco consumption is not only harmful for oral health but also leads to several non-communicable and systemic diseases. NCDs and dental caries are chronic and preventable conditions with a bidirectional relationship implicated by modifiable major risk factors such as tobacco consumption. Decreasing the consumption of tobacco use may improve oral health and reduce the risk of the development of NCDs. Also, regular dental visits should be scheduled to monitor the oral health status of factory workers. Additionally, tailored intervention for tobacco cessation should be implicated to maintain the general and oral health of industrial workers.

## Introduction

The era of industrialization has evolved over the years, and there has been continuous transformation of urbanization and development. The industrial landscape has diversified through the promotion of local manufacturing of products and investment in exportation. Sambhal City, located in India, is known widely for its handicraft items made up of bones and horns from carcasses or dead animals. According to a nationwide survey census 2011, Sambhal district had the lowest average literacy rate (48.28%) in India compared to the national average (74%). However, it is less than 100 miles away from the national capital, New Delhi [1]. As the literacy rate is lowest, most of the population depends on agriculture and daily wages such as food-vending, labor work, etc.

There are various types of industries, such as menthol and iron and steel factories. Still, the production of bone items is more widespread, from small factories to industrial mills situated within the vicinity of the city. In the bone factories, workers remove bones from keratin horns of carcasses and cut them into pieces of various sizes. These small pieces go for washing in refinery machines for cleaning. Then segregation started as large and medium-sized pieces were filtered out for handicraft materials and small pieces for fertilizers and animal foods. There are various products made up of animal’s horns (pots, combs, artificial jewelry, dressing buttons) and bone products (game dice, inlay boxes and trays, table lamps) etc.

Lifestyle, behavior and habits influence health in various occupations, and symptoms of oral diseases might be the first indication of any occupation-related illness. Their initial detection may help in identifying such conditions and thereby prevent them. Several occupations can predispose the workers to develop oral diseases because of the nature of their work. This condition can be due to distinct occupational factors that might occur because of exposure to chemical, organic, or inorganic substances [2]. Kanerva et al. reported that oral and pharyngeal ulcers, sloughy lesions, and bleeding presented due to exposure to different types of acids among blacksmiths and conjunctivitis due to the deposition of gold fumes in the eyes among goldsmiths. Also, ulceration of fingers, gingivitis, and loss of teeth due to arsenic exposure, a well-known carcinogenic element, has been reported among various factory workers [3].

Dentists and physicians need to identify the oral manifestations of diseases regarding various occupations. Timely diagnosis will not only help screen cases originating from relevant occupation but will also prevent further obstacles to those working in such conditions. Studies reported that grinding surfaces of teeth tend to retain dust particles that may lead to attrition and abrasion among cement workers and occupational fractured teeth among miners [4–6]. These studies also revealed that dental caries, missing teeth, periodontal conditions, erosion, and grinding were the most common oral health problems among factory workers.

Oral health is an integral part of general health and impacts individuals’ well-being. Periodontal conditions (gum diseases) and non-insulin-dependent (Type-II) diabetes mellitus have a bidirectional relationship. Patients with periodontal conditions are four times higher risk of having Type-II diabetes mellitus [7]. Another study found that oral diseases are linked with general health and correlated with cardiovascular diseases, preterm birth, and pneumonia [8].

In 2022, WHO released an Oral Health Status Report and noticed that more than half of the global population is affected by at least one of the oral diseases, dental caries being the most common disease with the highest burden in India. Additionally, trends of oral diseases showed that the estimated incidence of oral diseases has increased by 1 billion new cases globally from 1990 to 2019, of which 632 million people are affected in India [9]. Although the estimated prevalence of untreated dental caries of permanent teeth is 28.8% in India, children and youth are at risk population [10]. Prevalence of metabolic disease as Type-II diabetes mellitus was higher (41%) among people with gum diseases such as periodontitis in the Indian population [11]. Also, tooth loss has a significant association with hypertension among the older population and may lead to cardiovascular diseases [12].

The majority of oral and non-communicable diseases share most of the common non-biological risk factors, mainly unhealthy diets and sweetened beverages, tobacco, and alcohol consumption. However, WHO reported the premature mortality rate of NCDs as 86% and the highest-burden mostly in low-and middle-income countries [13]. WHO’s Global Action Plan for NCDs emphasized undercutting these causative factors to prevent non-communicable diseases to achieve the Sustainable Development Goals (SDG 3.4) till 2030 [14]. In response, on March 2023, World General Health Assembly passed a resolution to reduce the burden of oral diseases and general health cannot be achieved without oral health as oral health is integrated with prevention and control of NCDs under the umbrella of Universal Health Coverage [14]. On 15^th^ December 2023, WHO recognized Noma or Cancrum oris, a gangrenous stomatitis as a neglected tropical disease and prevalent globally among children with malnutrition and weakened immune system [15].

Among industrial workers, burnout may take a toll on health due to coping habits of work stress, missing family etc. Frequently, occupational stress may lead to substance abuse, alcohol and tobacco consumption. A multi-centric study done among various industrial workers reported that the prevalence of tobacco consumption is higher than that of the general population and the most common habit to relieve work stress among industrial workers [16].

However, the correlation between dental caries and sugar has already been documented in previous literature, as sugar is the primary substrate that promotes an acidic environment for cariogenic bacteria [17]. There is a lack of a relationship between tobacco consumption as a risk factor for the development of dental caries and other oral diseases. To the best of our knowledge, based on a literature search, this is the first study to assess the association between oral health status and tobacco as a major risk factor among bone-factory workers.

## Material & methods

This cross-sectional study was conducted among 500 study participants aged under 20 years and above with a minimum of 20 teeth present, who gave written informed consent, and who worked in a bone factory. Participants less than 20 years of age were excluded following WHO Guideline Basic Oral Health Surveys (1997) to record the Decayed Filled Missing teeth (DMFT) index for adults [18]. Additionally, the mean age of complete eruption of third molars or wisdom teeth is 19 (2.56) years, due to which inflammation may affect the accurate measurements of soft tissues [19]. Participants who were pregnant and lactating women, children, workers having any chronic health conditions or comorbidity, and intellectually disabled people were excluded from the study as these conditions also influence the actual measurements of oral health status of individuals. DMFT index not only records the decayed or missing teeth but also previous dental caries treatment failures such as recurrent caries and missing or lost teeth due to dental caries.

We took permission to conduct the study priorly from the IERB (Institutional Ethical & Review Board) Kothiwal Dental College and Research Centre (Certificate no.—KDCRC/IERB/12/2020/03). Also, we carried out a pilot survey that pretested the clinical examination and survey questionnaire among 27 bone-factory workers.

The codes and criteria for the diseases and conditions were observed and recorded in the WHO Oral Health Survey- Basic Performa-1997. The examiner and recorder were priorly calibrated and trained by recording oral health Performa and interview questionnaires among the patients. The same subjects were examined again by another examiner for the reliability of the investigator. The investigator calibration was done to ensure the uniform interpretation, understanding, and application of the survey procedures and to make a consistent judgment of clinical examination. The intra-examiner reliability was assessed using Cohens Kappa statistics (α = 0.90) for WHO Performa. The data recorder was trained to record clinical and interview variables before conducting the survey (α = 0.89). Initially, training was done for three consecutive days, and after a gap of five days to imbibe the methods, the calibration was rechecked (Kappa α = 0.90).

The study was conducted from September 2021 to March 2022 in the study area. The interview schedule was made in English, translated to Hindi as a regional language, and again translated into English to check for errors. We scheduled a systematic survey to examine estimated workers according to the convenient time of the factory workers and authorities available for study schedules in the bone factories.

### Study population

All bone factories had about the same environment in terms of ventilation of rooms, hygiene, dry crushed powder, and dust particles scattered around the machines, floor, and over the clothes, face, and other parts of the body of bone-factory workers in the study area.

### Sample size

The sample size was obtained by using the expected proportions of oral health diseases to be 50%, assuming the margin of error to be 0.05 at a confidence level of 95%, and the sample size was calculated to be 384. Considering a non-response rate of 10%, the sample size was determined to be 423. Though 423 subjects were needed for the study, we also examined 77 other bone-factory workers who were willing to get examined from selected bone factories due to limited access to oral healthcare services in the area and who gave consent to participate in the study and present at the time of the survey in bone-factories. The study population was homogenous as all clinically examined and interviewed participants were bone-factory workers and working in the same selected bone factories from a homogenous population within the study area.

### Sampling method

Multistage Stratified Cluster sampling was done in four stages to recruit study participants. We estimated a sampling frame minimum of 8000 bone-factory workers distributed in the study area. Sambhal District was divided into eight administrative blocks. We selected five blocks randomly as strata in different geographical regions. In the first stage, out of 27 quarters, 18 quarters or wards (three urban, five rural, and seven peri-urban) as primary sampling units were listed independently from five blocks. Bone factories were distributed away from an urban population due to the requirement of large field areas and the unpleasant smell of bones of carcasses. In the second stage, we randomly selected five clusters of bone factories as secondary sampling units from 18 wards. In the third stage, sixteen bone factories were randomly chosen from five subclusters in the district. In the fourth stage, five bone-factories were randomly selected and 100 participants were randomly recruited from each five bone factories in the study area.

### Clinical examination

The instruments for clinical examinations were sterilized in an autoclave before being carried out to the bone factories in ample amounts (thirty sets of plane mouth mirrors and probes) to continue ongoing examinations of subjects. We used WHO Oral Health Surveys–Basic Methods Performa (1997) for intra-oral examination. A single examiner assessed the oral health conditions of the subjects to avoid inter-examiner bias using the Type III American Dental Association method for natural light. The subjects sit upright on an ordinary chair with a headrest facing natural daylight. The examiner stood to the subject’s right while the trained data recorder was seated on the left side of the participants. Each form was checked by the examiner at the end of the day to ensure accuracy. All the oral examinations were done in the bone-factory premises in order to make it feasible for every worker to participate in the study.

The time taken for clinical examination was recorded as twenty minutes and seven minutes for the interview questionnaire for each participant. Clinical examinations include correctly identifying the number of decayed, missing and filled teeth. Instruments used in the clinical examination include a WHO-specified Community Periodontal Probe (CPI) and a plane mouth mirror [18]. Oral examination was done in the bone factory premises to make it convenient for the study participants as workers do not have to lose their working hours and are available for the study schedule. Thus, a total of 500 bone-factory workers employed in the bone factories were recruited by Multistage Stratified Cluster sampling to undergo oral health assessment using the WHO Oral Health Survey-Performa for intra-oral and extra-oral examination and interviewed using pre-structured modified WHO-STEPWISE survey tool [20]. In an interview, participants were asked questions regarding their socioeconomic details, duration of employment, oral health-seeking behavior, frequency of teeth cleaning, material used to clean teeth, and brushing techniques. Questions related to tobacco consumption habits were assessed mainly: do you consume tobacco? If yes, which type of tobacco (smoking/SLT/dual), type of smoking or smokeless tobacco (SLT) product used for chewing tobacco, frequency of tobacco uses a day, the total number of tobacco products used in any form, since how long have been consuming tobacco, when did you start using tobacco in any form, status of current tobacco use [active smokers (whether daily or occasional) or former smokers].

### Statistical analysis

We entered the data in Microsoft Excel 2019 (Microsoft Corporation, USA), and missing data were omitted in any case. The data were entered into STATA MP 17 (STATA Corp, USA), and analysis was carried out with what remained. Descriptive statistics were calculated for sociodemographic data, including dependent and independent variables. The outcome variable was oral health status and covariates include sociodemographic variables, tobacco habits, and oral hygiene-seeking behavior-related variables. Brinkman index was constructed from frequency of tobacco consumption multiplying with duration of tobacco use for severity level of tobacco users. Bivariate variables were calculated by Chi-square test. We used Multivariate Regression analysis for the DMFT index and oral mucosal lesions. A significant *p*-value was set less than 0.05 with a 95% confidence interval (CI).

## Results

This cross-sectional study was conducted among 500 bone-factory workers with ages ranging from 20 to 64 years. The total number of males was 342 (68.40%), while 158 (31.60%) were females. The mean (SD) age of participants was 33.18 (10.7) years, and mean DMFT score was 2.84 (3.12) of factory workers. The majority of the participants 275 (55%), were in the younger age group of 20–32 years and had the lowest mean DMFT score of 2.28 (2.65). However, 66 (13.2%) participants aged 46–64 years had the significantly highest mean DMFT score of 5.38 (4.91) [*p* < 0.001].

Participants with a higher income and education (graduate and above) had significantly favorable mean DMFT scores of 1.72 (±1.72) and 1.98 (2.14), respectively, in comparison to workers with low or no education and less income [*p*<0.001] shown in "Table 1". Among the bone-factory workers, production workers had the highest mean DMFT score of 3.08 (3.43).

**Table 1:** Distribution of DMFT index based on socioeconomic characteristics among the bone-factory workers. N = 500.

Characteristics	Frequency n(%)^a^	DMFT Index	*p-*value^b^
**Sex**			
Male	342 (68.40)	2.95 (3.43)	0.001*
Female	158 (31.60)	2.56 (2.80)	
**Age (years)**			
20–32	275 (55)	2.28 (2.65)	<0.001*
33–45	159 (31.80)	2.74 (2.83)	
46–64	66 (13.20)	5.38 (4.91)	
**Education level**			
No education	54 (10.8)	3.15 (3.27)	<0.001*
Primary & middle	266 (53.2)	3.25 (3.56)	
High School	130 (26)	2.20 (2.75)	
Graduate & above	50 (10)	1.98 (2.14)	
**Occupation type**			
Labor	345 (69)	3.08 (3.43)	0.001*
Admin staff	101 (20.2)	2.70 (2.94)	
Others	54 (80.1)	1.48 (2.12)	
**Geographical area**			
Urban	170 (34)	2.23 (2.82)	<0.001*
Peri-urban	259 (51.8)	3.20 (3.36)	
Rural	71 (14.2)	2.93 (3.62)	
**Income level (monthly)**			
Poorest	351 (70.2)	3.15 (3.40)	0.002*
Poorer	112 (22.4)	2.18 (3)	
Richer	37 (7.4)	1.72 (1.72)	
**TOTAL**	500	2.84 (3.12)	

^a^Frequency expressed as n (%), DMFT index was expressed in mean (standard deviation).

^*b*^*p* value was calculated by using the Chi-square test.

*Significant level < 0.05.

Prevalence of intra-oral lesion was 34% among factory workers, of which most common oral-lesions were Oral Submucous Fibrosis (OSF) 47 (9.40%), followed by leukoplakia 29 (11%). Also, out of every 100 participants, 12 workers presented extra-oral lesions on the face, head & neck, and facial surface among factory workers shown in "Table 2". On clinical examination, tobacco users had significantly more intraoral (56%) and extra-oral (15.41%) lesions in comparison to non-tobacco users shown in “Table 2”.

**Table 2:** Distribution of precancerous lesions presented based on tobacco use among bone-factory workers. (N = 500).

Characteristics	Frequency (%)	Tobacco-users	Non-tobacco users	*p* value^a^
**Intra-oral lesions**				
No abnormality	330 (66)	115 (43.23)	215 (91.88)	< 0.001*
Leukoplakia	29 (5.8)	29 (10.9)	0	
Lichen planus	3 (0.60)	3 (1.13)	0	
Ulceration	15 (3)	12 (4.51)	3 (1.28)	
Candidiasis	20 (4)	18 (6.77)	2 (0.85)	
Dental Abscess	10 (2)	6 (2.26)	4 (1.71)	
Severe Smokers’ Palate	22 (4.40)	18 (6.77)	4 (1.71)	
Oral submucous fibrosis	47 (9.40)	44 (16.54)	3 (1.28)	
Others	24 (4.80)	21 (7.89)	3 (1.28)	
Total	500	266 (53.2)	234 (46.8)	
**Extra-oral lesions**				
Normal Condition	441 (88.20)	225 (84.59)	216 (92.31)	0.005*
Ulceration (head, neck)	42 (8.40)	28 (10.53)	14 (5.98)	
Ulceration (nose, cheeks)	10 (2)	10 (3.76)	0	
Others	7 (1.40)	3 (1.13)	4 (1.71)	
Total	500	266 (53.2)	234 (46.8)	

^a^*p* value was calculated by using Chi-square test.

Regarding occupational characteristics, 41% of bone factory workers reported complaints of abnormal dry mouth, of which 11% had severe an abnormal dry mouth condition, very often. Interestingly, 66% of bone-factory workers with abnormal dry mouth did not present any oral lesions. Regarding tobacco consumption, 142 (53.38%) workers significantly had abnormal dry mouth were tobacco users and 81 (34%) were non-tobacco users (p<0.001). We also observed that compared to others, increasing frequency of tooth-brushing and using toothpaste will significantly decrease the odds of unfavorable DMFT Index shown in “Table 3”. It should also be noted that Table 3 includes unadjusted findings and was used to construct the final adjusted model for regression analysis.

**Table 3:** Unadjusted linear regression for DMFT based on characteristics among study participants. N = 500.

Characteristics	Crude OR	CI .95		*p* value^a^
		Upper	Lower	
**Sex**	Ref: female			
Male	0.68	0.37	1.26	0.217
**Age**	Ref: 33–45			
20–32	1.58	0.86	2.89	0.140
46–64	22.17	6.77	14.11	<0.001*
**Education**	Ref: no education			
Primary	1.10	0.43	2.83	0.841
High School	0.38	0.14	1.07	0.067
Graduated & above	0.31	0.09	1.08	0.20
**Occupation**	Ref: Labor			
Admin	0.68	0.33	1.40	0.296
Others	0.20	0.08	0.51	0.001*
**Income**	Ref: poorest			
Poorer	0.38	0.19	0.75	0.006*
Richer	0.24	0.08	0.72	0.011*
**Duration of work**	Ref: < = 5 years			
> 5 years	2.76	1.54	4.95	0.001*
**Dry mouth** (yes)	6.13	3.53	10.66	<0.001*
**Teeth cleaning frequency**	Ref: once a day			
Two or more a day	0.51	0.25	1.05	0.068
2–6 times a week	1.87	0.79	4.46	0.155
Once a week	48.54	4.37	539	0.002*
**Materials used for teeth cleaning**	Ref: other materials			
Toothpaste	0.34	0.19	0.59	<0.001*
**Pain or infection**	Ref: not present			
Present	37.71	8.39	169.47	< 0.001*
**Dental visit**	Ref. never visited			
Visited in last five year	12.84	7.58	21.73	<0.001*

^a^OR, Odds Ratio. Ref. Reference.

^b^*p* value were calculated by using Chi-square test.

*Significant level < 0.05.

**Fig 1:**
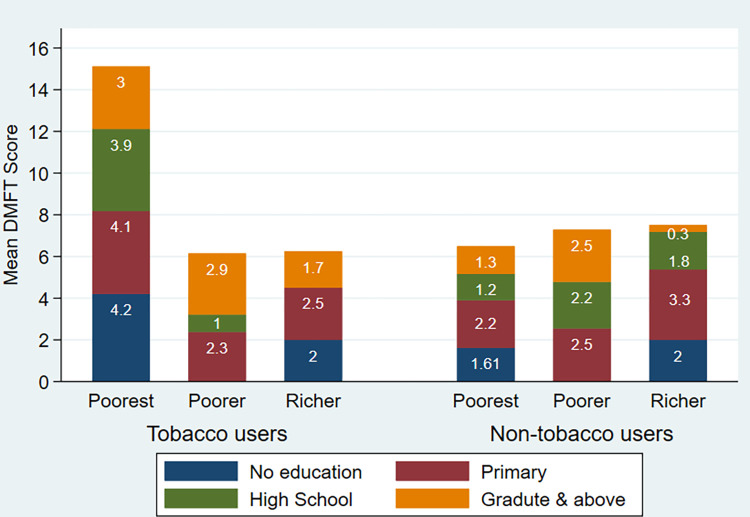
DMFT index based on education and income among bone-factory workers.

Prevalence of tobacco users was 53.2%, of which 18% were smokers, 30.2% were SLT users, and 5% were dual users among the factory workers. Poorest tobacco users with no education had the unfavorable DMFT index 4.20 (2.35) [Fig 1].

**Fig 2:**
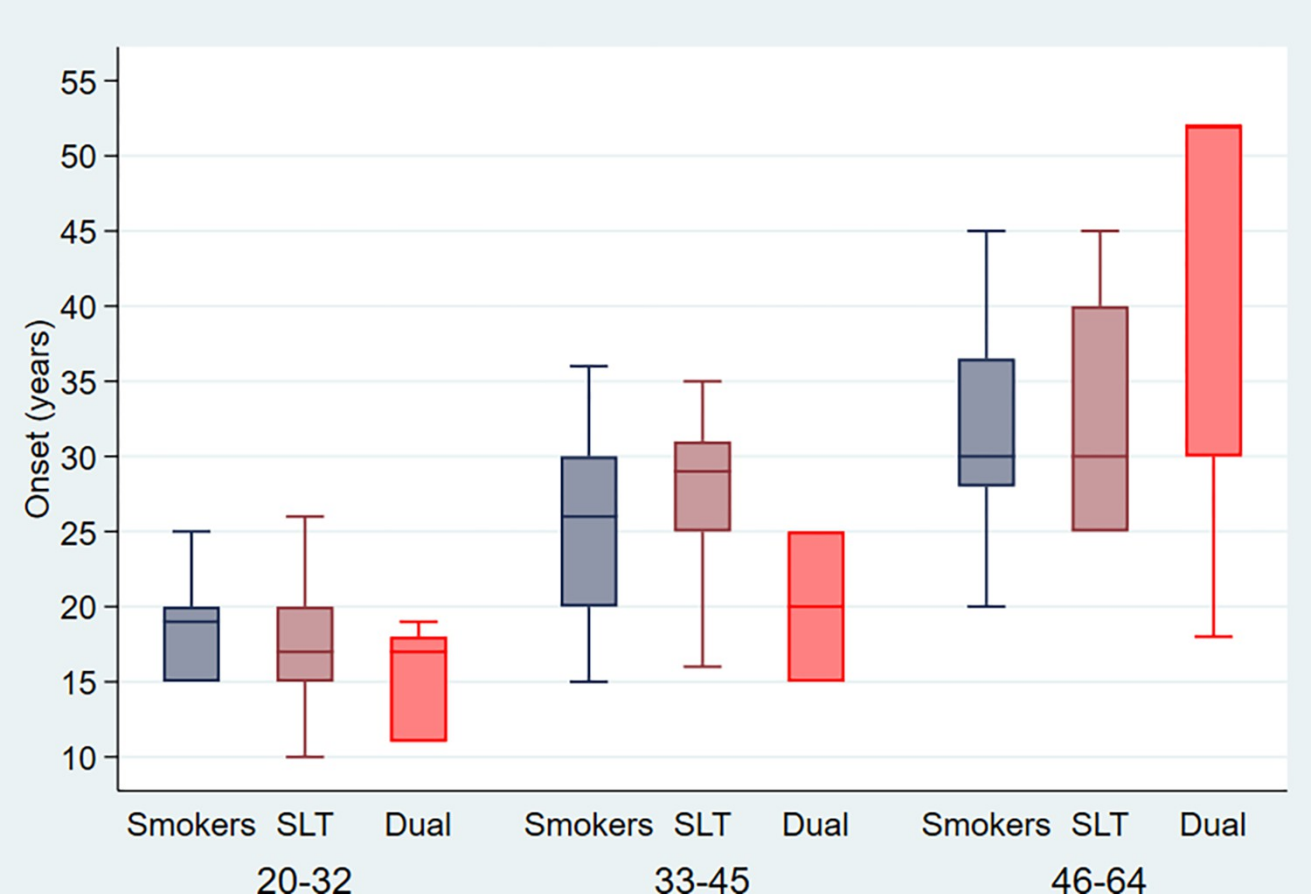
Distribution of tobacco users based on age of onset among bone-factory workers.

Minimum age of onset of tobacco use was ten years, the interquartile range (IQR) was 22 years, and the median age was 15 years only among bone-factory workers. The younger age group had the highest prevalence 130 (48.87%) of tobacco users among the workers. Also, younger tobacco users started using tobacco before 18 years, with a mean age of 15.25 (3.61) years. Also, the initiation age of all dual tobacco users in the younger age group was below 20 years [Fig 2].

In bivariate regression analysis regarding tobacco habits, tobacco users were significantly 4.57 increased odds and SLT users were 7.70 higher odds of harmful DMFT Index compared to non-tobacco users (p<0.001). Brinkman index exhibits that compared to non-tobacco users, mild tobacco users had 4.11 (COR 4.11CI.95: 2.31–7.24, *p*<0.001*)* higher odds and moderate users were 15.42 (COR 15.42 CI.95: 3.74–63, *p*<0.001) increased odds of critical DMFT score shown in "Table 4".

**Table 4:** Unadjusted linear regression results for tobacco users based on DMFT index. N = 266.

Characteristics	N (%)	DMFT Index	Crude OR	CI .95	*p* value^a^
**Tobacco use**					
Non-tobacco users	234 (46.8)	2.02 (± 2.50)	Reference		
Tobacco users	266 (53.2)	3.55 (± 3.64)	4.57	2.61–7.98	<0.001*
**Type of tobacco (**ref: non-tobacco users)					
Smokers	90 (33.83)	2.64 (± 2.88)	1.84	0.85–3.94	0.119
SLT users	151 (56.77)	4.06 (± 3.72)	7.70	4.04–14.64	<0.001*
Both	25 (9.4)	3.68 (± 4.97)	5.23	1.43–19.13	0.013*
**Tobacco onset age in years (**ref: non-tobacco users)					
< = 18	86 (32.33)	3.09 (± 3.35)	2.87	1.31–6.29	0.008*
>18	180 (67.67)	3.77 (± 3.76)	5.70	3.08–10.56	<0.001*
**Current Status** (refer: non-tobacco user)					
Daily Active users	227 (45.4)	3.23 (± 3.51)	3.35	1.89–5.93	<0.001*
Occasional or < daily	39 (7.8)	5.35 (± 3.95)	28.03	9.70–80.99	<0.001*
**Brinkman Index (**ref: non-tobacco-users)					
Light users	479 (95.80)	2.74 (3.22)	4.11	2.31–7.24	<0.001
Moderate users	21 (4.20)	4.76 (3.5)	15.42	3.74–63	<0.001

DMFT, Decayed Missing Filled Teeth. Ref, Reference. OR, Odds Ratio. (SD), Standard Deviation. CI, Confidence Interval. SLT, Smokeless Tobacco.

^a^Column percentages were calculated.

^b^*p* value calculated using the Chi-square test.

*Significant level < 0.05.

In adjusted multivariate analysis, regarding the age of workers, the odds of unfavorable DMFT score was 8.68 times (AOR 8.68 CI.95: 4.04–18.64, *p*<0.001) higher, and for oral mucosal lesions, more than 2.5 folds increased odds (AOR 2.57, CI.95: 1.43–5.84, *p* = 0.003) for elder age-group compared to younger workers holding other variables constant.

However, compared to the poorest workers, the odds of DMFT score of richer workers is 60% (AOR 0.39 CI.95 0.15–1.02, *p* = 0.056) fewer and for oral lesions were about significantly 78% (AOR 0.22 CI.95: 1.04–3.59, *p =* 0.014) fewer compared to poorest workers holding other variables constant.

Regarding tobacco consumption behavior, compared to non-tobacco users, a higher mean DMFT score of 3.55 (3.64) with 3.52 times higher odds of harmful DMFT Index (*p = 0*.*020)* and 2.79 (CI.95: .84–9.21, *p <* .*001*) higher odds of developing oral lesions were observed among tobacco users holding other variables constant (Table 5).

**Table 5:** Adjusted multivariate regression analysis of characteristics influencing oral health status of bone-factory workers. N = 500.

Characteristics		DMFT Score			Oral Mucosal Lesions		
		**AOR** ^**a**^	**P-value**	**CI**^**b**^ **.95**	**AOR** ^**a**^	**P-value**	**CI .95**
**Age**	20–32	1			1		
	33–45	1.10	0.726	0.63–1.93	1.52	0.110	2.55–2.55
	46–64	8.68	< 0.001*	4.04–18.64	2.88	0.003*	1.43–5.84
**Income**	Poorest	1			1		
	Poorer	0.69	0.236	0.37–1.28	2.02	0.026*	1.09–3.74
	Richer	0.39	0.056	0.15–1.02	0.22	0.014*	0.07–0.73
**Tobacco Use**	No	1			1		
	Yes	3.52	0.045*	1–12.35	2.79	< 0.001*	0.84–9.21
**Brinkman Index**	Non-tobacco users	1			1		
	Mild users	1.75	0.372	0.51–5.97	6.80	0.001	2.22–20.81
**Teeth cleaning material**	Others	1			1		
	Toothpaste	0.56	0.028*	0.33–0.94	1.05	0.834	0.64–1.73
**Visit Dentist**	Never	1			1		
	≤ 5 years	9.10	< 0.001*	5.55–14.92	0.61	0.042*	0.38–0.98
**Dry mouth**	No	1			1		
	Yes	3.40	< 0.001*	2.06–5.61	1.89	0.008*	1.18–3.03
**Constant**	Beta	2.42	0.002*	1.37–4.27	0.05	< 0.001*	0.03–0.10

^a^AOR, Adjusted Odds Ratio

^b^CI, Confidence Interval.

*Significant level < 0.05.

In our study, the correlation between tobacco consumption with DMFT Index and oral-mucosal lesions was 26.14% and 51.24%, respectively; on the contrary, the correlation between DMFT Index and sweets or other sugary diets was 2.69% only.

Regarding Brinkman Index, mild tobacco users were 75% (AOR 1.75 CI.95: 0.51–5.97, *p =* 0.372) higher odds of high DMFT score compared to non-tobacco users though this was not statistically significant. Compared to non-tobacco-users, odds of developing oral lesions among mild tobacco-users was significantly 6.80 (CI.95: 2.22–20.81, *p<*0.001) times higher holding all other variables constant.

Regarding oral hygiene habits, toothpaste users were significantly 44% (AOR 0.56 CI.95:.033–0.94, *p* = .028) fewer odds of high DMFT Index compared to those factory workers who use other materials for brushing teeth holding all other variables constant.

Bone-factory workers who had ever dental visits were significantly highest odds 9.10 (CI.95:5.62–15.02) of critical DMFT for ever-visitors compared to never-visitors (*p* <0.001). Also, the odds of developing oral lesions were significantly decreased by 41% for dental visits, holding other variables constant (*p =* 0.029).

Bone-factory workers with abnormal dry mouth were significantly 3.40 (CI.95:2.06–5.61) higher odds of high DMFT Index and oral mucosal lesions. The model also predicts that workers with abnormal dry mouth have 1.89 (CI:1.18–3.03, *p* = 0.008) increased odds for developing mucosal lesions in comparison to workers without abnormal dryness of mouth holding all other variables constant.

Overall, the results suggest that elder age, income, tobacco use, dentifrices such as brushing materials, dental visits, and abnormal dry mouth were significantly associated with the hard tissue lesions (dental caries) and soft tissue lesions (oral mucosal lesions) of the oral cavity among bone-factory workers.

## Discussion

The selective occupational characteristics, including exposure to dust particles of bones to this population, make the comparison with other epidemiological studies precautious about the study’s description. Bone fertilizer is a well-known source of infection in animals and often in men [21]. Fumes of the fertilizer become airborne and may enter the oral cavity, leading to various oral diseases. However, this is the first documented study to assess the risk factors for oral health status of bone-factory workers in India’s least literate city, Sambhal.

In our study, one-third of female bone-factory workers (31.6%) presented compared to male participants. This could be due to social and cultural norms in the community, as females are generally considered for household chores, caretakers and staying at home. According to the International Labor Organization (ILO-2019) report, there is a trend of gender disparity in terms of employment among labor workers, as female employment has dropped to 32% since 2003 [22]. However, there is no difference between DMFT scores among males and females, which coincides with a study done by Mittal et al. in 2020 [23]. The mean age of bone-factory workers was 33.18 (10.7) years, which coincides with the study done by Batista et al. [24].

We also observed that socioeconomic conditions impact oral health, as higher income and educated groups have a lower prevalence of oral diseases. Another study done by Moradi et al. also observed the lowest DMFT score in the wealthiest group [25]. This condition could be because poor and uneducated workers have less access to and utilization of oral healthcare services in India. A systematic review done by Talukdar et al. in 2022 reported that only 23% of the Indian population has utilized dental healthcare services in both the public and private sectors in the last decade [26].

Compared to poorest workers, other workers with better socioeconomic status (SES) have the privilege to access to healthcare services. Additionally, illiterate and poorest tobacco users have a critical mean DMFT score. Low SES and adverse habits are the deadly combination to poor general and oral health. This fact suggests that oral and general health screening and effective health education programs should reach out to marginalized population regularly to prevent diseases in the community.

Labor workers had a significantly higher DMFT Index than administrative staff in bone factories. This could be explained by occupational exposure to bone dust particles in the oral cavity, leading to dental diseases among labor workers. Similar findings were observed in other studies among sugar mill workers and industrial factory workers [2,27]. On the contrary, this condition was not the same with solid waste collectors [28]. This suggests that various occupational exposures have a different impact on oral health. A healthy lifestyle and oral & personal hygiene can prevent several oral and general diseases.

Intraoral lesions result from insulting oral mucosa by physical, chemical, thermal, and biological causative agents. Tobacco induced oral lesions develop due to thermal and harmful chemical toxins. Among the bone-factory workers, the most common precancerous lesion was Oral Submucous Fibrosis (OSF), which is caused by consumption of smokeless tobacco (SLT) and manifests trismus or reduced mouth-opening. A study done by Kommapati et al. also reported that most common tobacco-product was SLT and most common lesion was OSMF among industrial workers [29]. This could be due to the cultural acceptance of chewing tobacco in South Asia and India, second to ranking in the prevalence of SLT. Additionally, among tobacco users, SLT is considered less harmful compared to smoking form of tobacco [30].

The second most common oral mucosal lesion, Leukoplakia, is caused by smoking-attributable tobacco (SAT) consumption. Smoking not only manifests oral precancerous conditions but also invites several NCDs such as hypertension, cardiovascular diseases, COPD and asthma which can be prevented by changing habits and lifestyle. A study done by Norvinda et al. among workers reported that ulcers are associated with socioeconomic status and lifestyle of individuals [31].

Most common oral problem self-reported by bone-factory workers was abnormal dry mouth. This condition could either be due to physiological, pathological or, drug-induced etc. However, pathological dry mouth or xerostomia could be an occupational hazard among factory workers due to the deposition of bone dust particles in the mouth. A study by Raj et al. in 2016 observed that dry mouth was more than four times higher among battery workers exposed to acid fumes that form and charge the batteries compared to non-exposed control group (packing etc.) [32].

The prevalence of tobacco consumption was higher among bone-factory workers, which coincides with a study done by Akram et al. among industrial workers [33]. Younger workers were exposed to tobacco earlier and felt the euphoria of nicotine. Interestingly, in our study, the minimum age of onset of tobacco consumption was at the 10^th^ birthday of a bone-factory worker, and the mean age of initiation for dual tobacco users was 12.30 (12) years, which coincides nearly with a study done by Prashar et al. among construction site workers [34]. This issue should be addressed with a behavioral change awareness campaign for education among youth. Also, one in every ten bone-factory-worker consumes both forms of tobacco as dual tobacco users, which is similar to the findings reported by Epperson et al. among youth [35]. A study reported that male dual tobacco users are at higher risk of gestational cancer and pregnant women for gestational hypertension [36].

Tobacco not only affects the health of individuals but also the environment, such as pollution and resources, i.e., water, electricity, land and workforce etc. are dissipated, which could be utilized to grow foods & grain crops to deal with food security [37].

There is an association between onset and factors for initiation of tobacco consumption. Interestingly, in our study, the mean age of tobacco initiation was much lower for smokeless tobacco compared to smokers, which raises serious health concerns regarding the health of youth. There are several factors of tobacco use in different populations to initiate tobacco consumption at an early age. This could be due to easy access to smokeless tobacco (gutkha, pan-masala, and khaini or snus, etc.), passing the time with the continuation of work, peer pressure and curiosity to try out tobacco. Ansari et al. also observed that the introduction of tobacco by the family, pleasure, peer pressure, advertisements, and promotions in media were the main reasons for initiating tobacco consumption among power loom workers [38].

In our study, the majority of tobacco users reported pleasure was the main driving force to consume tobacco in any form. In the market, the flavoring tobacco is sold on the name of sweeteners supari (a crushed betel nut) pan-masala by sellers and promoted by tobacco companies that attract the youth with curiosity to try it once. These nicotine dependence strategies should be discouraged in the community to save the youth from diseases like cancers. Global Adult Tobacco Survey (GATS, 2016–17) reported similar findings regarding the age of initiation for SLT use and a significant difference in early consumption of mean initiation for SLT compared to smoking [39].

The prevalence of dual users is 5.3% in India which is lower compared to Bangladesh (8.7%) in South Asia [40]. The proportion of dual tobacco users in India may be lower, but the number of people at risk is highest in the South Asia region. This may be due to the unintended and ineffective capability of tobacco cessation programs and policies in India. Also, the Cigarette and Other Tobacco Products Act (COTPA, 2003) was amended in 2020 but not implemented strictly with uniformity in all states of India [41]. Also, smokers usually shift to SLT to control their smoking habit gradually, which may lead them to be dual users often double the risk of diseases. A study also reported that those who were using snus or pouch tobacco did not use SLT until they quit smoking.

Tobacco consumption in any form increases the risk of higher odds of cardiovascular diseases such as hypertension and brain strokes [42]. Anti-tobacco programs and policies should ban the advertisement and endorsement of tobacco products to prevent the further burden of oral and non-communicable diseases. Brinkman Index demonstrate the severity level of the tobacco consumption. There is an association between dose and response relationship of tobacco use as moderate users were at higher risk of critical DMFT Index.

In our study, the multivariate model predicted that the odds of critical DMFT Index were ten folds higher among elderly bone-factory workers with the highest mean DMFT Index, which coincides with the study done by Bommireddy et al. [43]. Additionally, a survey by Ngo et al. among adults and elders in 2018 observed that mean DMFT scores increased with aging [44]. Also, increasing age affects salivary glands adversely, hypofunction decreases quality and quantity of saliva that results in decreased salivary buffering capacity for cariogenic acids that dissolve the layers of the tooth and lead to dental caries [45]. Older people are the most vulnerable to oral and dental diseases that hinder the quality of life.

There is a disparity regarding DMFT Index in socioeconomic class. This could be due to limited access to oral health, busy with work, and, most importantly, income constraints. Increasing per thousand units of workers’ income decrease odds for richer workers compared to poorest and poorer workers. Similarly, odds of oral lesions are higher among poorest and poorer than richer workers. As per Moradi et al., socioeconomic status will decrease the odds of higher mean DMFT 0.19 times the odds in higher income groups [25].

This model also predicted that being a tobacco user has more than three times odds of having a higher mean DMFT score than the odds of being a non-tobacco user. Tobacco consumption in any form threatens general health, oral soft and hard tissues. In a metanalysis, the strong correlation was found between tobacco consumption and dental caries as well [46]. A per Mittal et al. mean DMFT score was higher among tobacco users in comparison of non-tobacco users [23].

In our study, odds of oral-lesions were associated with tobacco consumption and tobacco habits affected adversely soft tissues more than double folds compared non-tobacco-users after adjustment in multivariate models. This could be due to the fact that oral soft tissue mucosa is more sensitive to toxins and chemicals. As per Kaur et al., every fifth worker had oral-precancerous lesions and every fourth worker presented oral cancer clinically and most commonly used tobacco products were SLT among textile workers [47].

Tobacco companies promote the exposure of tobacco products in media and activities such as free samples, coupons and gift-vouchers to attract youth and students. This should be closely monitored and banned to prevent nicotine dependance among young tobacco users. Early exposure of tobacco induced morbidity and mortality has been well documented in the literature [48]. Community outreach and effective support is essential to increase awareness about harmful effects of tobacco and promote healthy habits among workers.

In bivariate analysis SLT users were more than seven times higher odds of harmful mean DMFT score compared to non-users. Similarly, Doddawad et al. reported that prevalence of harmful DMFT Index were higher among smokeless tobacco users (mainly gutkha chewers) compared to other tobacco products [49]. On contrary to this, a study done by Chaudhury et al. among general population in India, observed a positive correlation between DMFT Index and SLT use but negative correlation with smoking and suggested that smoking attributable tobacco contains thiocyanates which has anticariogenic properties [50]. A study done by Koul et al. observed that cariogenic bacterial count was more prevalent in tobacco users than non-tobacco users and SLT users were more at increased risk of higher mean DMFT score in comparison of smokers and non-tobacco users [51]. Thats a debatable issue that findings of these studies regarding type of tobacco consumption vary in different populations.

Highest odds of oral-lesions were presented among light-tobacco users compared to non-tobacco users. This finding suggests the positive dose response relationship between tobacco use and oral-lesions. As per Sellappa et al. higher proportion of cariogenic bacteria (Streptococcus Mutans) were profound (48%) among tobacco users with increased numbers of bacteria among SLT users (54%) than non-tobacco users [52]. Mittal et al. also observed that increase frequency and duration of tobacco use also increases the mean DMFT score.

Odds of dental lesion (caries) is highest compared to oral-lesion. This is due to the fact that outer layers of oral mucosa (epithelium) are relatively more permeable, rich in blood vascularity and more sensitive to chemicals and thermal damage that cause inflammation. Outer layer of dental hard tissues as enamel are formed by various minerals contents and directly resistant to thermal and chemical changes [19].

In multivariate model, regarding frequency of sweets consumption and oral health seeking behavior such as, brushing techniques and frequency for teeth cleaning have no statistically significant difference among bone-factory workers. This condition could be because the variance of DMFT Index could be explained by 3% by these covariates if other variables are constant in this population (R^2^ = 0.03). This is due to the fact that other characteristics or variables have higher effect on DMFT than oral hygiene habits. Other studies have also reported that these variables have no significant impact on DMFT [23,53]. This finding shows the importance of tobacco cessation (AOR 3.52) and highlights the fact that oral hygiene practice only is not adequate to prevent dental and oral mucosal lesions among factory workers. Although, individuals should maintain oral hygiene to prevent inevitable oral diseases.

Regular Dental visits had highest odds of DMFT Index due to interventional treatment among factory workers. Nearly, half of the population (49.20%) had dental decay presented at the time of survey. Only 14% bone factory workers had restoration as filled with or without decay of teeth. This could be because of lack of dental care habits and majority of the workers going for interventional measurement for treatment needs only rather than oral prophylaxis. This issue highlights the urgency of preventive measures such as oral prophylaxis, health education about oral hygiene, behavioral change counselling, fluoridation and vouchers for treatment etc. in community campaigns. Ngo et al. observed that compared to dental visits more than three months, initial dental visitors who visited the dentist less than three months have significant lower mean DMFT score [44].

Strength of this study is predicting the severity of impact of various characteristics on oral mucosal and dental tissues after adjustment of variables among bone-factory workers. Also, there was a paucity in the literature regarding several variables such as exposure of bone-factory environment, frequency, duration and age of initiation or onset of tobacco use etc. We included these variables into models to assess the effects of variables of oral health status among bone-factory workers in the present study. Limitations include smaller sample size and systemic health assessment of bone-factory workers. So that results should be disseminated carefully. Further prospective cohort studies with larger sample size should be conducted to assess the incidence rate of dental caries and general health among bone-factory workers.

## Conclusion

Overall, bone factory workers had a poor oral health status. Tobacco consumption is a primary risk factor for oral mucosal and dental lesions. Further added to the tobacco consumption adversely impacted on oral health status among bone factory workers. Regular dental and medical checkups should be organized by the owners of factories that may improve the oral health status of bone-factory workers. However, maintaining and promoting oral health may be an early life intervention also to prevent NCDs and to reduce the double burden of oral and non-communicable diseases on healthcare system. Medical and dental health rights of factory workers as mentioned in ESI Act 1948 should be implemented and practiced in the industries. Further studies are required to assess the general and oral health regarding occupational exposures among the bone-mill workers. Tailored policy interventions should be acquainted for the overall health of factory workers. This study urges the dialogues among policy makers and program managers to tailor the prevention of non-communicable designs with implemented inclusivity of oral health.

## Supporting information

S1 Fig Mean DMFT score based on education and wealth index among bone-mill workers. (TIF)

S2 Fig Distribution of tobacco users based on age onset of tobacco use. (TIF)

S1 File Interview schedule. (PDF)

S2 File Dataset. (DTA)
