# Supplementary figures and images for: Impact of tobacco habits on poor oral health status among bone-factory workers in a low literacy city in India: A cross-sectional study
Source: PLoS One. 2024 Apr 17;19(4):e0299594. doi: 10.1371/journal.pone.0299594 (PMC11023192; doi:10.1371/journal.pone.0299594)

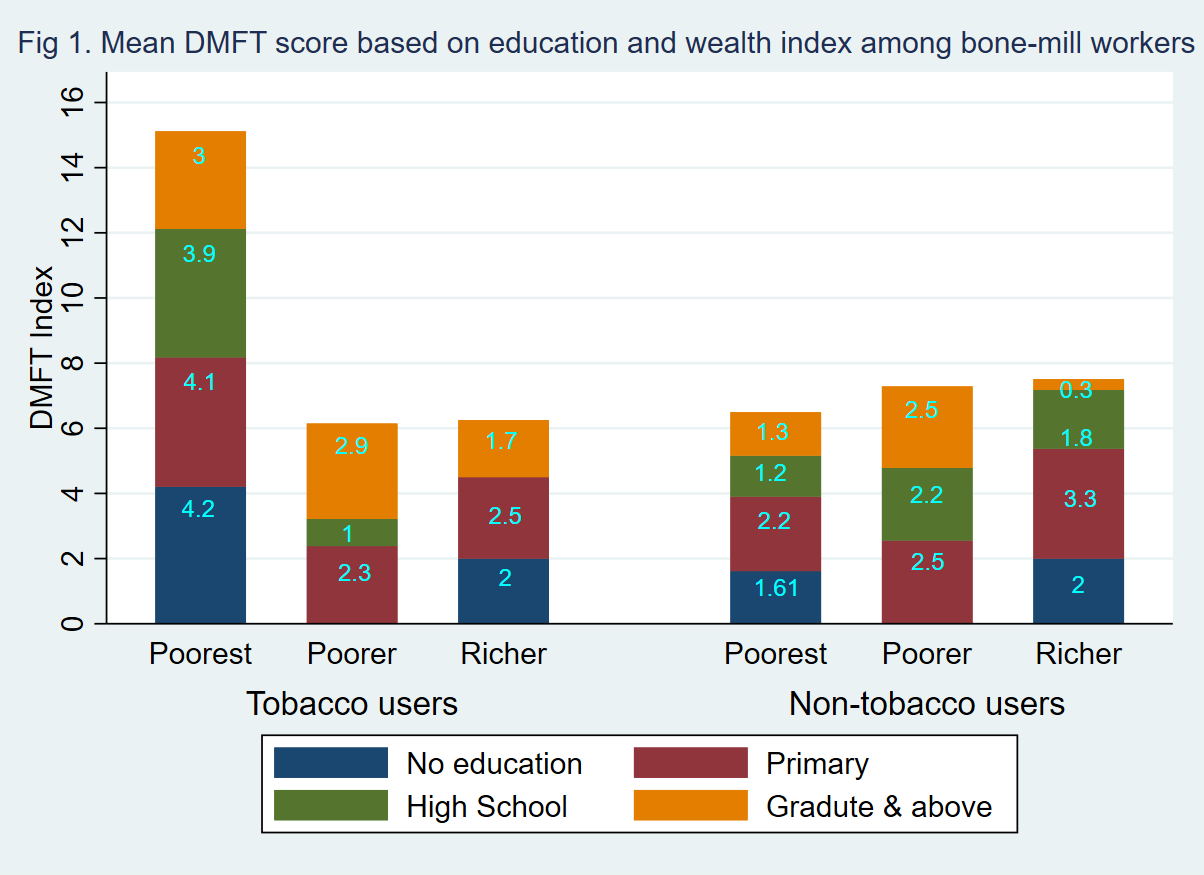

Supplement: S1 Fig — (TIF) [file pone.0299594.s001.tif]

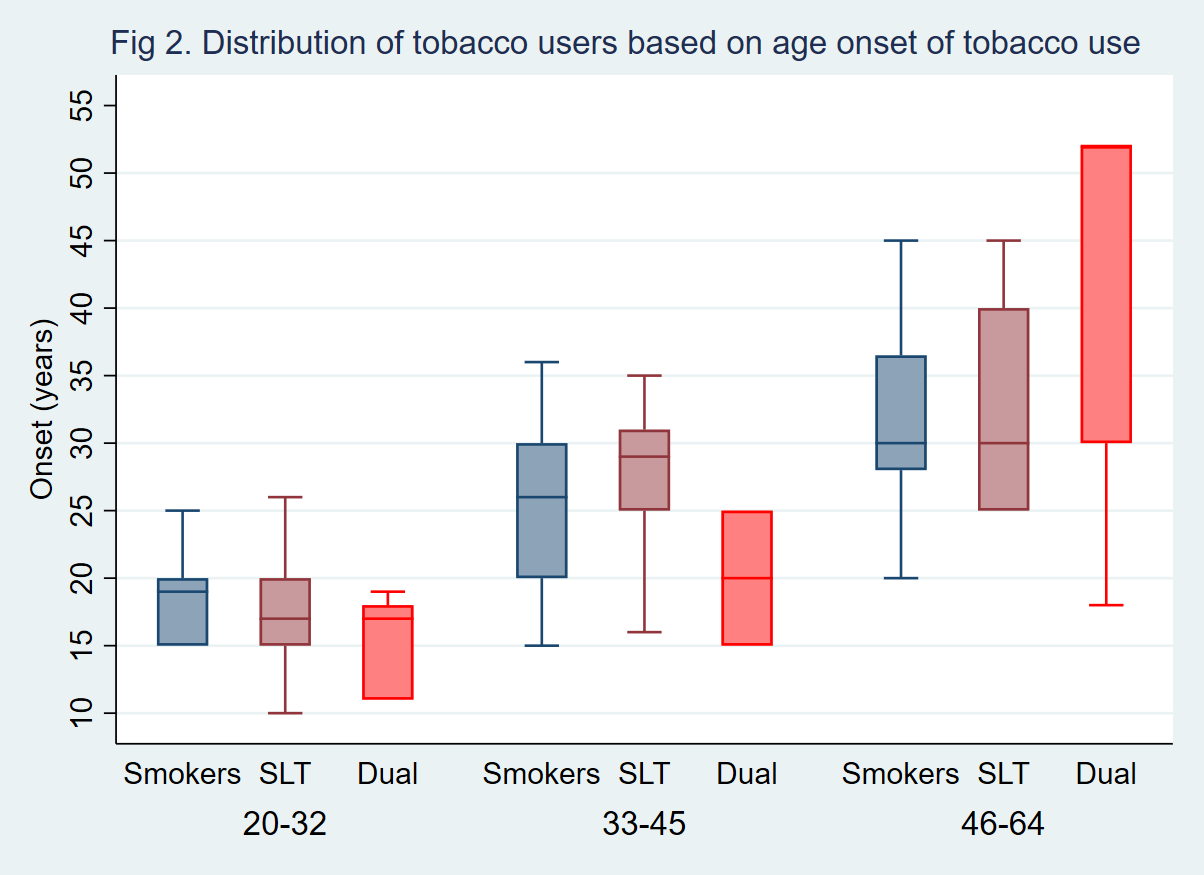

Supplement: S2 Fig — (TIF) [file pone.0299594.s002.tif]
