# Supplementary material for: Impact of tobacco habits on poor oral health status among bone-factory workers in a low literacy city in India: A cross-sectional study
Source: PLoS One. 2024 Apr 17;19(4):e0299594. doi: 10.1371/journal.pone.0299594 (PMC11023192; doi:10.1371/journal.pone.0299594)
Supplement: S1 File — (PDF) [file pone.0299594.s003.pdf]

INTERVIEW SCHEDULE

Section 1: Socio-economic details

1) Marital status : a) Married b) Unmarried c) Divorced/ Separated d) Widow/Widower
2) Duration of employment:

Section 2: Oral Related Personal Habits

1. How often do you clean your teeth?
1) Once a day
2) Two or more times a day
3) 2 to 6 times a week
4) Once a week

2. What do you use to clean your tooth?
1) Tooth paste
2) Tooth powder
3) Chew stick/wooden twig
4) Thread/ dental floss
5) Charcoal/Ash
6) Toothpicks (wooden/plastic)
7) Finger with tooth-powder/paste

3. What is your brushing technique?
1) Horizontal
2) Vertical
3) Both horizontal and vertical
4) Rotary
5) Don't know

4. How often do you eat or drink any of the following foods, even in small quantities? (Read each item and tick appropriately)

{Several times a day=6 ; Every day=5; Several times a week =4; Once a week=3; Several times a month=2; Seldom/never =1}

	6	5	4	3	2	1
1) Fresh fruit..	☐	☐	☐	☐	☐	☐
2) Biscuits, cakes, cream cakes.....................................	☐	☐	☐	☐	☐	☐
3) Sweets, pies, buns...	☐	☐	☐	☐	☐	☐
4) Honey or jam	☐	☐	☐	☐	☐	☐
5) Lemonade, Coca Cola or other soft drinks.................	☐	☐	☐	☐	☐	☐
6) Tea or coffee with sugar..	☐	☐	☐	☐	☐	☐

Section 3: Tobacco and Alcohol related Habit

5. Do you use tobacco:
- Yes / No ....

6. If yes then which type of tobacco do use:
1) Smoking attributable
2) Smokeless

7. If smoking, then what type of products consumed:
1) Cigarettes
2) Bidi
3) Hand-rolled
4) Hookah
5) Others.. (please specify)

8. If smokeless, then what type of smokeless tobacco:
1) Khaini
2) Gutkha
3) Paan
4) Zarda
5) Others.............................

9. How many times in a day do you take tobacco:
1) <5 times
2) 5-10 times
3) > 10 times

10. At what age did you start using tobacco (any form)?
-

11. Number of tobacco products smoked per day? (If smoke >one tobacco product)
-

12. How long have you been using tobacco?
-

13. Do you currently use tobacco on daily basis?
1) Daily
2) Occasional
3) Never
4) Former (mention time when did you quit) ....

14. Which of the following factors enhance your need to use tobacco? ***(Ask only current or past tobacco users)***

1) Stress due to the workload
2) Missing family and close friends
3) Poor relation with staff/employ
4) The lack of alcohol/narcotics
5) Habitual
6) Addictive behaviour
7) Feeling pleasure/satisfaction
8) Keep awake at night
9) Other………………………………………………………………………….(*please give the examples*)

15. Have you ever attempted to quit smoking? (*Please, give the number of quit attempts*).

1) Yes, but only at liberty (……………….times)
2) No

16. Please, check the reasons for quitting attempts:

1) Limited access to cigarettes.
2) The will to save some money.
3) The fight against your own weakness.
4) Somebody else's instigation
5) The will to gain an authority
6) Advertising
7) Other …………………………..
8) Consider it as harmful

17. Do you consume alcohol?

1) Yes 2) No

18. If yes,

1) Duration of alcohol use (since when).....................................
2) Frequency of alcohol intake……………………..................

Section 4: Oral Health Care Seeking Behaviour

19. Do you have any pain or discomfort in your mouth in past one year?

1) Yes
2) No
3) Don't Know

20. How would you describe the state of your teeth and gums? Is it "(1) Excellent ", "(2) Very good", "(3) Good", "(4) Average", "(5) Poor", or "(6) Very poor"

- Teeth…………..…………............ Gums…………..…..........

21. When did you last visit a dentist? (If answer to question is never visited skip to Q. 23)

1) Less than 6 months
2) 6–12 months
3) More than 1 year but less than 2 years
4) 2 years or more but less than 5 years
5) 5 years or more
6) Never visited a dentist

22. What was the reason of your last visit to the dentist?

1) Consultation/advise
2) Pain or trouble with teeth, gums or mouth
3) Treatment/ follow-up treatment
4) Routine check-up/treatment
5) Don't know/don't remember
6) Never visited

23. Because of the state of your teeth or mouth, how often have you experienced any of the following problems during the past 12 months?

	Very Often (4)	Fairly Often (3)	Some times (2)	No (1)	Don't know (0)
1) Difficulty in biting foods..................................	☐	☐	☐	☐	☐
2) Difficulty chewing foods.......................................	☐	☐	☐	☐	☐
3) Difficulty with speech/trouble pronouncing words.	☐	☐	☐	☐	☐
4) Dry mouth...	☐	☐	☐	☐	☐
5) Felt embarrassed due to appearance of teeth……….	☐	☐	☐	☐	☐
6) Felt tense because of problems with teeth or gums..	☐	☐	☐	☐	☐
7) Have avoided smiling because of teeth............ ……….	☐	☐	☐	☐	☐
8) Had sleep that is often interrupted............ ……….	☐	☐	☐	☐	☐
9) Have taken days off work…………….	☐	☐	☐	☐	☐
10) Difficulty doing usual activities........... ……….	☐	☐	☐	☐	☐
11) Have reduced participation in social activities............ ……….	☐	☐	☐	☐	☐
